# The Anti-Arthritic and Immune-Modulatory Effects of NHAG: A Novel Glucosamine Analogue in Adjuvant-Induced Arthritis

**DOI:** 10.1155/2013/487610

**Published:** 2013-07-18

**Authors:** Syed Uzair A. Shah, Huma Jawed, Shahid I. Awan, Shazia Anjum, Shabana U. Simjee

**Affiliations:** ^1^H.E.J. Research Institute of Chemistry, International Center for Chemical and Biological Sciences, University of Karachi, Karachi 75270, Pakistan; ^2^Dr. Panjwani Center for Molecular Medicine and Drug Research, International Center for Chemical and Biological Sciences, University of Karachi, Karachi 75270, Pakistan

## Abstract

Rheumatoid arthritis (RA) is potentially devastating condition which lacks good treatment options. Pro-inflammatory cytokines interleukin-1beta (IL-1**β**), tumor necrosis factor-alpha (TNF-**α**), and oxidative stress markers such as nitric oxide (NO) and peroxide (PO) are mediators of RA pathogenesis. In the present study N-[2,4,5-trihydroxy-6-(hydroxymethyl) tetrahydro-2H-pyran-3-yl]acrylamide (NHAG), analogue of glucosamine, was evaluated in adjuvant-induced arthritic model of rats. The disease progression was monitored by analysing arthritis scoring, loss of body weight, paw oedema, and histological changes in joints. RA associated hyperalgesia was evaluated by gait analysis. The serum or plasma levels of NO, PO, glutathione (GSH) superoxide dismutase (SOD) IL-1**β** and TNF-**α** were analyzed to monitor the state of disease severity. The arthritic control animals exhibited significant increase in arthritic score (*P* < 0.003) and paw oedema (*P* < 0.001) with parallel loss in body weight (*P* < 0.04). The NHAG-treated arthritic animals exhibited refinement in the gait changes associated with arthritis. NHAG also significantly decreased the NO (*P* < 0.02) and PO (*P* < 0.03) with concurrent increased in GSH (*P* < 0.04) and SOD (*P* < 0.007). Both IL-1**β** (*P* < 0.001) and TNF-**α** (*P* < 0.001), were significantly decreased in NHAG-treated group. Thus NHAG might have a therapeutic potential for arthritis by exerting antioxidative and immunomodulatory effects.

## 1. Introduction

Rheumatoid arthritis is a chronic, progressive, and systemic inflammatory disease, characterized by synovial proliferation and joint erosions [1, 2]. Nonsteroidal anti-inflammatory drugs (NSAIDs) are used as an important part of therapeutic regime to suppress the pain and inflammation associated with RA [3, 4]. Although NSAIDs are very effective in minimizing RA-associated symptoms, its beneficial effect is strongly associated with severe side effects such as gastrointestinal complications, renal failure, and hepatic toxicity [5–7]. NSAIDs treatment has another important disadvantage that they have no impeding effect on disease progression and do not have any protective role in tissue or joint injury [8]. Furthermore, clinical studies have shown that long-term use of NSAIDs in arthritis can enhance the joint destruction and also inhibit the synthesis of glycosaminoglycans [9]. In addition to these classical available therapies, there are several reports regarding the use of disease-modifying antirheumatic drugs (DMARDs) and anti-TNF therapy, which act as potentially effective therapies for rheumatoid arthritis [10, 11]. DMARD treatment is currently based on symptomatic relief of pain and inflammation associated with arthritis to increase joint function and mobility. In spite of greater potency as anti-inflammatory and antiarthritic agents over other treatment regime, the DMARDs are associated with side effects such as renal or hepatic cirrhosis, muscular weakness interstitial pneumonitis, fatal agranulocytosis, aplastic anaemia, severe myelosuppression, and toxic epidermal necrolysis [11–13]. 

Effective and potential treatment of rheumatoid arthritis could be those agents that can protect the synovial fluid and cartilage for further destruction [14]. Chondroprotective agents are extensively used and their positive outcomes in protecting and regenerating cartilage and maintaining healthy joint function are extensively reported [15, 16]. Glucosamine is one of the chondroprotective agents that enhance development of chondrocytes, synovial fluid, and joint cartilage degradation [17, 18]. Glucosamine is a structural component of cartilage and connective tissues and is required for the syntheses of glycosaminoglycans [19]. It suppresses the production of matrix metalloproteinases, collagenases, and phospholipase [20]. Studies have shown that during the development and progression of arthritis, the synthesis of glucosamine becomes defective thereby resulting in articular degeneration [20]. Glucosamine has beneficial effects when given as supplement in osteoarthritis, rheumatoid arthritis [21, 22]. Supplemental glucosamine can reverse the joint degradation and articular function [23]. Glucosamine rebuilds the cartilage by incorporating itself in the synthesis of proteoglycans and glycosaminoglycans [24], and it also inhibits the synthesis of prostaglandin E2, production of reactive oxygen species, proinflammatory cytokines (IL-1*β* and TNF-*α*) by activated neutrophils and other immune cells [25, 26]. Although glucosamine is good natural therapy for osteoarthritis and rheumatoid arthritis that have effective anti-inflammatory and antiarthritic activity of glucosamine, there are some side effects that are associated with the use of glucosamine such as the allergic reaction to its source, that is shellfish; another side effect is to change the insulin regulation and alter blood sugar level so it is contraindicated in diabetic patients, mild gastrointestinal symptoms [27, 28]. Furthermore, due to complex mechanism of joint pain and destruction, glucosamine alone is sometimes not enough so it is important to further improve its biological activity. 

Our research group has taken an interest in the synthetic manipulations of amino sugars to develop some efficient derivative of glucosamine by using the Boullanger strategy [29]. Earlier we have reported that one of the **β**-D-glucosamine derivatives NHAG that is (6-hydroxy methyl-3-(1-methylene-allylamino)-tetrahydro-pyran-2,4,5-triol) demonstrated effective antiarthritic activity in collagen-induced arthritic model [30]. The present study was designed to identify antiarthritic effects of NHAG in adjuvant-induced arthritis (AIA) and its effects on the circulatory levels of proinflammatory cytokines IL-1*β* and TNF-*α* and oxidative stress markers glutathione, nitric oxide, and peroxide. Since NHAG is a novel compound and we could not find any related studies done elsewhere using this compound therefore, at this stage we have discussed its observed activities in light of the studies done on the glucosamine (parent compound). 

## 2. Materials and Methods

### 2.1. Animals

Female Sprague-Dawley rats weighing 200–250 g were housed at regulated temperature (21° ± 2°C) and humidity controlled room (55 ± 5%) under pathogen-free conditions on a 12/12 h light-dark cycles with the free access to standard laboratory diet and water. The ethical guidelines of International Association for Study of Pain in conscious animals [31] and the guidelines set by the Scientific Advisory Committee on Animal Care, Use, and Standards, International Center for Chemical and Biological Sciences (ICCBS) were followed.

### 2.2. Adjuvant/Drugs/Reagents

**Figure 1:**
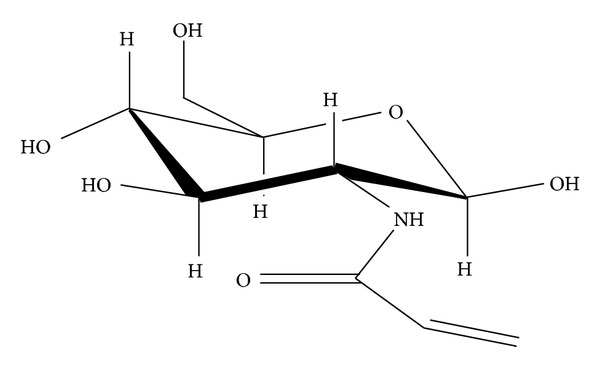
Structure of NHAG (N-[2,4,5-trihydroxy-6-(hydroxymethyl) tetrahydro-2H-pyran-3-yl]acrylamide) synthesized from a parent compound glucosamine.

The lyophilized heat-killed* Mycobacterium tuberculosis* MT H37Ra (DIFCO Laboratories, Detroit, USA) was used as an adjuvant for the induction of arthritis. Indomethacin (Sigma Chemicals, USA) was used as a reference drug. NHAG, a *β*-D-glucosamine derivative (Figure 1), was synthesized by our chemist collaborator at ICCBS, University of Karachi, Pakistan, using the Boullanger strategy [29].

### 2.3. Induction of Arthritis and Drug Treatment

Animals were randomly distributed in the control and test groups with 12 animals in each group. The complete Freund's adjuvant (CFA) was prepared by mixing lyophilized MT H37Ra in mineral oil. Volume of 0.1 mL of 1 mg suspension of MT H37Ra was injected intradermally at the base of the tail using a sterile hypodermic needle under anaesthesia with a combination of ketamine/xylazine in the dose of 20 mg/kg/5 mg/kg (i.p). Treatment was initiated on the same day of arthritis induction. Following the arthritis induction, the animals were closely monitored by the author and the technical staff who observed animals daily until the full blown arthritis was observed. The treatment regime followed for NHAG and indomethacin throughout this study is outlined in Table 1.

### 2.4. Clinical Assessment of Adjuvant-Induced Arthritis

Rats were evaluated on alternate days for arthritis using a macroscopic scoring system, where 0 = no signs of arthritis, 1 = swelling and/or redness of the paw or one digit, 2 = two joints involved, 3 = more than two joints involved, and 4 = severe arthritis of the entire paw and all digits [32]. The arthritis severity score for each rat was calculated by adding the scores for each individual paw. The clinical severity of arthritis was also determined by quantifying the change in the body weights and paw inflammation (as an indicator of oedema). Paw inflammation was quantified based on paw swelling and histological changes. Since the paw inflammation can be presented as a change in the hind paw volume, the paw volume was therefore measured by using a Plethysmometer. The body weight and hind paw volumes were measured in both the control and test groups on day 0 and then on alternate days until day 18 when the experiment ended.

### 2.5. Measurement of Nociception through Gait Analysis

Nociception associated with arthritis was assessed by measurement of gait parameters using TreadScan System (Clever Sys. Inc., USA) to record the gait of the animals. The working of this apparatus is based on an original design of Clarke [33] that was developed for analyzing the gait development in young rats. The data was collected by placing an individual rat in the chamber, recording its spontaneous movement across the central position. Complete, non hesitant transits of the 30 cm central section were used for analysis. The apparatus is equipped with video recorder that records the animal's movement in the chamber. The software provided with this system (TreadScan) can analyze the video and determine various characteristic parameters that are related to the pathophysiological conditions. Various postural and kinematics metrics of gait dynamic were determined by dissecting the time of each limb spent in various portion of walking phase. The parameters measured in this study are stance time (paw in contact with the floor), swing time (paw in the air), stride length, and running speed. The gait recorded in the control and test groups at the beginning of an experiment was used as the baseline reading (day 0).

### 2.6. Assessment of Articular Damage/Histological Examination

At the end of each experiment, animals were humanely sacrificed by decapitation, and specimens were taken from knee joints for histological examination. The joints samples were fixed in 10% formalin and then placed in decalcifying solution (10% EDTA solution) for two weeks. The decalcification solution was changed twice every week. After decalcification, the samples were processed, embedded, cut into 10 *μ*m sections, and stained with haematoxylin and eosin (H & E). The severity of the arthritis in knee joint was scored numerically; that is, the pathology was rated 0 to 5, with 0 being normal and 5 being the greatest extent and degree of involvement [34, 35].

### 2.7. Determination of Oxidative Stress Parameters

#### 2.7.1. Measurement of Reactive Oxygen Species (ROS)

The levels of biochemical markers of oxidative stress, that is, nitric oxide (NO) and peroxide (PO) were determined in the plasma samples of both normal and arthritic groups. Animals were sacrificed at the time of peak inflammation. Whole blood was collected through cardiac puncture and dispensed in the heparin-containing tubes. The heparinized blood samples were centrifuged and plasma was collected and used for determination of NO and PO using quantitative colorimetric assay kits, that is, the Quantichrom Nitric Oxide Assay kit and the Quantichrom Peroxide assay kit Diox-250 (BioAssay Systems, USA). The manufacturer directions were carefully followed. The samples were run in duplicate in a 96-wells plate, and the plate was read at 540 nm. The Quantichrom Peroxide assay kit Diox-250 system is an improved method which utilizes the chromogenic Fe^3+^-xylenol orange reaction in which a purple complex is formed when Fe^2+^ provided in the reagent is oxidized by peroxides present in the sample. The intensity of the colour measured at 540 nm is an accurate measure of the peroxide level in the samples.

#### 2.7.2. Measurement of Glutathione (GSH) and Superoxide Dismutase (SOD)

The concentration of glutathione was determined in plasma using the colorimetric Quantichrom Glutathione assay kit (Bioassay Systems) that determines the reduced form of glutathione. The samples were run in duplicate in a 96-well plate. The optical density measured at 412 nm at the end of the experiment is directly proportional to glutathione concentration in the sample.

Superoxide dismutase activity was measured in plasma using Superoxide Dismutase Assay Kit (Cayman Chemical Company, USA). The manufacturer's protocol was carefully followed. Briefly, 2 mL of blood was collected by cardiac puncture from anesthetized animal to get 1 mL of plasma. The assay was performed in 96-well plates. The absorbance was measured at 450 nm using ELISA plate reader. The average absorbance of each standard and sample was calculated. The linear rate (LR) was determined for all standards and samples following the manufacturer's directions. The linearized SOD standard rate (LR) was plotted as a function of final SOD activity (U/mL). 

### 2.8. Quantitative Analysis of Proinflammatory Cytokines IL-1*β* and TNF-*α*

The proinflammatory cytokines IL-1*β* and TNF-*α* were quantified in serum samples by enzyme-linked immunosorbent assay (ELISA) using Rat TNF-*α* and IL-1*β* ELISA Kit (Komabiotech, South Korea). The plates were read at 450 nm on ELISA plate reader. The standard curve was generated by plotting the absorbance of each TNF-*α* and IL-1*β* standards against its concentration. The levels TNF-*α* and IL-1*β* in serum samples were determined from standard curves using absorbance reading at 450 nm.

### 2.9. Statistical Analysis

To analyse the data, the Statistical Package for the Social Sciences (SPSS) software was used. The values are represented as means ± S.E.M. to describe the data in figures. The data were analysed using one-way analysis of variance (ANOVA). Furthermore, the Bonferroni's post hoc test was used to determine which group mean differs. Values below the level of 0.05 were considered as significant.

## 3. Results

### 3.1. Clinical Assessment of Adjuvant-Induced Arthritis

**Figure 2:**
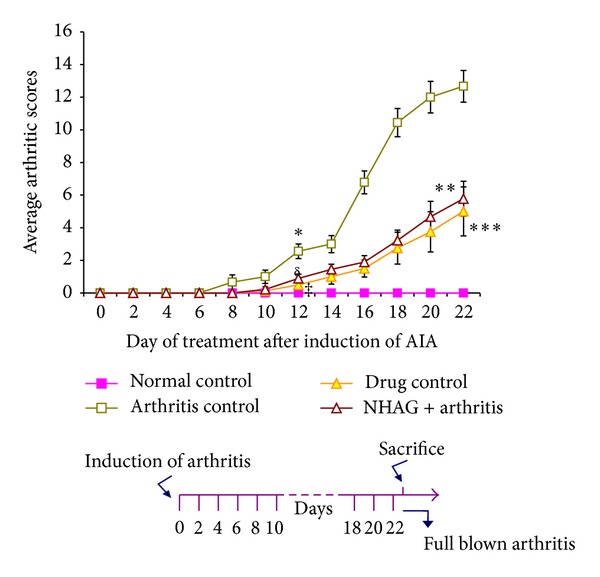
Onset of clinical signs of arthritis in the arthritic control group. Each value represents the mean arthritic score ± SEM (*n* = 12/group). The arthritic score in arthritic control group was significantly higher compared to normal control animals (*P < 0.001) on day 8 onward. The indomethacin (^*δ*^ *P* < 0.001) and NHAG (^‡^ *P* < 0.02) treated groups exhibited a significant decrease in arthritic score on day 12 onward compared to arthritic control and continued till end of experiment (***P < 0.001 and **P < 0.003), respectively. The difference between NHAG and drug control groups was not significant. Although NHAG treatment was unable to bring the score near to the normal control groups, however, when compared to arthritic control group, the reduction was 60% (**P < 0.003).

The mean arthritic score for arthritic control group demonstrated evidence of clinical inflammation in one or both hind paws after day 8 (Figure 2). Initially erythema was observed in ankle joints, followed by involvement of the metatarsal and interphalangeal joints. Although the onset of disease symptoms in the treatment and drug control groups was delayed up to day 10, the disease was progressive, with joint recruitment following the same pattern, that is, tarsal, metatarsophalangeal, and then interphalangeal. The time course for the development and progression of disease, as assessed by the mean arthritic severity score, is shown in Figure 2. Mean arthritic severity score of 3 was evident in arthritic control rats on day 12 which was significantly different from normal group (*P* < 0.001). The bonferroni's post hoc test to find the difference between the various treatment groups revealed that the score of arthritic control group was significantly higher than the indomethacin- (*P* < 0.001) and NHAG-treated arthritic animals (*P* < 0.02) on day 12 and continued until the end of experiment. Our test compound was soluble in the saline; therefore, we tested saline as a vehicle control. No difference was found between saline treated and arthritic control animals. Statistical analysis also revealed no significant difference between these two groups; therefore it is not shown in the figures. In the arthritic and vehicle control groups, the incidence of disease was 100% at day 16 and remained as such until the end of experiment. In comparison to these control groups, a significant attenuation in the incidence of arthritis was observed with indomethacin (70%) and NHAG (60%) treatment.

**Figure 3:**
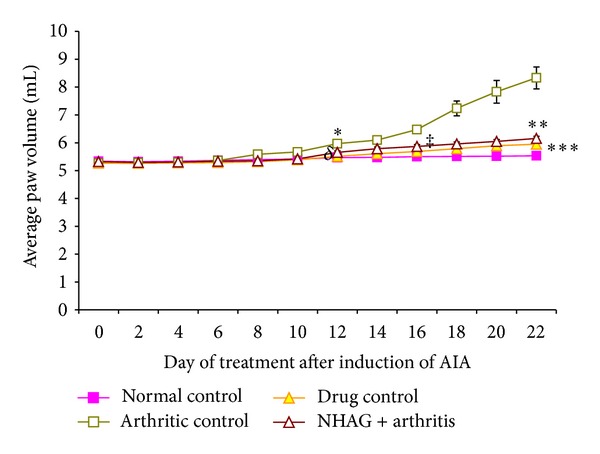
Effect of NHAG (5 mg/kg) on the average paw oedema in rats induced with arthritis. Each value represents mean paw oedema ± SEM of *n* = 12. The arthritic control group demonstrated a clear evidence of clinical inflammation in both hind paws from day 8 onward. The bonferroni's post hoc test showed that the paw volume of the arthritic rats was significantly higher than normal control group on day 12 onward (*P < 0.01). In comparison to the arthritic control group, a significant reduction in paw volume was observed in case of indomethacin (^*δ*^ *P* < 0.004) and NHAG (^‡^ *P* < 0.001) on days 12 and 16 and continued till end of experiment (***P < 0.001) and (**P < 0.001), respectively.

The clinical severity of arthritis was measured in terms of average paw swelling demonstrated in Figure 3. A slight increase was observed in the average paw volume of the normal control group over a period of 22 days which might be due to an increase in the normal body weight. In contrast, the arthritic control group demonstrated a clear evidence of clinical inflammation in one or both hind paws from day 8 onward. The analysis executed for different time frame used for measuring paw volume demonstrated that the significant difference between normal and arthritic control groups started on day 12 onward (*P* < 0.01) and continues to be significantly higher at the end of the experiment (*P* < 0.001). A mild increase in the paw volume was observed in the arthritic animals treated with NHAG (5 mg/kg) and indomethacin (5 mg/kg), but when compared to the normal group, this increase was found to be nonsignificant compared to normal control. Next, we compared the indomethacin- and NHAG-treated arthritic groups with the arthritic control animals. It was observed that both treatments reduced the inflammation as reflected by the measurement of paw volume. Statistical analysis also revealed a significant difference between arthritic control and indomethacin groups from day 12 onward (*P* < 0.004), whereas a significant difference was started from day 16 in case of NHAG- (*P* < 0.001) treated group. We also compared the treated groups and found no significant difference between indomethacin and NHAG treatments.

**Figure 4:**
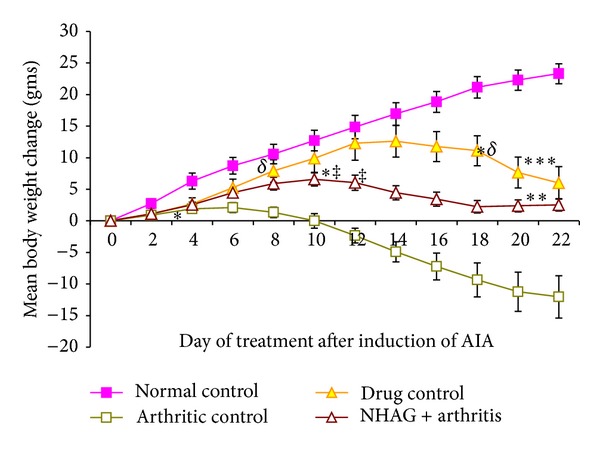
Mean body weight change over a period of 22 days after arthritis induction. Each valuerepresents the mean ± SEM of *n* = 12 per group. A gradual increase in the body weight of normal control group was observed throughout the course of 22-day experiment. The arthritic control group demonstrated a significant decrease in their body weight from day 4 (*P < 0.01) onward. When compared to normal control, the indomethacin- and NHAG-treated arthritic group showed a decline in their body weight from days 14 and 12, achieved significance on day 18 (^∗*δ*^ *P* < 0.04) and 12 (^∗‡^ *P* < 0.04), respectively. However, the reduction was significantly lower when compared to the arthritic control group on day 8 (^*δ*^ *P* < 0.008) and day 12 (^‡^ *P* < 0.03) and continued till end of experiment (**P < 0.04) and (***P < 0.004) for indomethacin- and NHAG-treated groups, resp.).

Another clinical manifestation of arthritis is the loss of body weight determining the severity of arthritis. The mean change in the body weight is shown in Figure 4. The body weights of the animals between the groups were not significantly different before commencement of the study. The statistical analysis revealed that the mean change in body weight of arthritic control group was significantly decreased (*P* < 0.01) on day 4 in comparison to the normal control group, and this reduction persists until the end of the experiment. On the other hand, the normal control and indomethacin-treated groups exhibited an increment in body weight up to day 14. However, from day 16, the mean change body weight started to decrease gradually in case of indomethacin treatment and this decrease was significant on day 18 onward (*P* < 0.04). In contrast, the mean change in body weight of NHAG-treated arthritic group started to decrease significantly from day 12 (*P* < 0.04); however, this decrease was stabilized on day 18, and no further body weight reduction was observed until the end of the 22-day experiments. Both indomethacin and NHAG treatments were next compared with the arthritic control group. It was observed that although a pattern of body weight reduction was seen in these treated groups, however, when compared to the arthritic control group, the reduction was significantly less in case of indomethacin (*P* < 0.008, on day 8) and NHAG treatment (*P* < 0.03, on day 12 onward).

### 3.2. Effect of NHAG on the Temporal Measurements of Gait

Means of individual gait parameters were not significantly varied among the groups when measured at the start of experiment. The mean of gait parameters including speed, stride length, stance time, and swing time observed on day 0 and day 20 is shown in the Table 2. 

The arthritic control animals showed a drastic decline in their speed as well as in their stride length which was significantly lower than the normal group (*P* < 0.001). In contrast, the arthritic animals receiving indomethacin and NHAG treatments exhibited a significant reduction in the mean speed on day 20 (*P* < 0.02 for drug control and *P* < 0.001 for NHAG) possibly because of slight inflammation in their paw at the end of the experiment. However, the stride length was found to be significantly increased in both the NHAG- and indomethacin-treated animals (*P* < 0.05). Next we compared the treated groups with arthritic control animals. A deficit in the mean velocity of indomethacin-treated animals was far less than the one observed in the arthritic control groups. One-way ANOVA analysis revealed a significant difference in the indomethacin and arthritic control groups on day 20 of the experiment (*P* < 0.02). When compared to the arthritic control group, the NHAG treatment showed refinement in the mean velocity, and statistically it was significantly higher from the arthritic control group (*P* < 0.03) on day 20. Regardless of the refinement we saw in the mean speed and stride length of the NHAG-treated group, the treatment was unable to completely reverse the deficit, and values were different from the normal group. 

The stance time and swing time of the arthritic control animals exhibited a significant increase on day 20 when compared to the normal animals (*P* < 0.001). Both treatments, that is, indomethacin and NHAG were unable to reduce the stance time; however, the increase in the measured parameter was significantly lower than the arthritic control group (*P* < 0.003 for indomethacin and *P* < 0.02 for NHAG-treated group). NHAG and indomethacin treatments markedly reduced the swing time on day 20 onward when compared to that of the arthritic control group (*P* < 0.05). In comparison to the normal group, the treated groups showed slightly nonsignificant increase in stance and swing time. 

### 3.3. Effect of NHAG Treatment on Joint Histology

**Figure 5:**
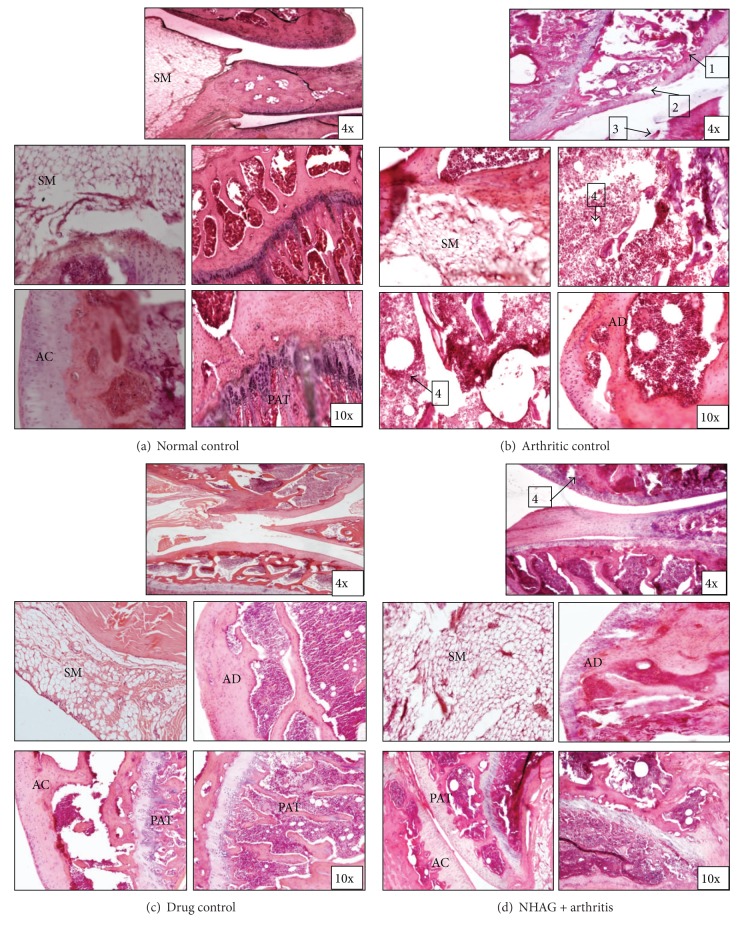
Histology of knee joints from arthritic treated, nontreated, and normal animals (hematoxylin and eosin staining). AC: articular cartilage; AD: articular disc; SM: synovial membrane; PAT: periarticular tissue. Normal control: note lack of lymphocytes infiltration of synovium and intact articular bone. Arthritic control: prominent lymphocytic infiltration of synovium with invasion of periarticular bone and vacuolization (arrow 1 and 4), collapse of articular surface, and articular bone destruction (arrow 2 and 3). Drug control: note mild infiltration of lymphocytes in synovium, however; the damage in articular bone is quite negligible. NHAG + arthritis: note mild to moderate infiltration of lymphocytes in synovium with slightly damaged articular bone (arrow 4).

The progression of disease was also monitored by joint histology. Representative joint histopathology of the groups is illustrated in Figures 5(a)–5(d). The histology of normal rat joints (Figure 5(a)) shows no inflammatory symptoms. The histological features of arthritic control group (Figure 5(b)) demonstrated a prominent proliferation of granulation tissue about the articular surface of the knee joints. Infiltration of lymphocytes and connective tissue replacement of destroyed cartilage was observed in case of the arthritic control tissues. The joint space was increased in comparison to normal control group. Within the treatment groups, indomethacin treatment demonstrated a marked reduction in the inflammatory changes; however, the degree of extent to which it reduces these changes was less compared to ones seen in the NHAG-treated arthritic group (Figure 5(c)). On the other hand, the joint histology of arthritic rats treated with NHAG (Figure 5(d)) revealed minimal evidence of inflammation or joint destruction in comparison to arthritic rats. In fact the synovial membrane in the joints was almost like normal synovium, with mild synovial hyperplasia.

### 3.4. Effect of NHAG Treatment on Oxidative Stress Parameters

Since saline was used as a vehicle for solubilizing NHAG therefore, we also administered this vehicle in the arthritic group. We did not found any effect of using saline as a vehicle on the following measured parameters, and the data was almost similar to that of the arthritic control group. Likewise, the data of NHAG treatment given to normal control did not exhibit any variation from the normal group. For the very same reason, the data of both these groups, that is, vehicle control and compound control group is not shown in the subsequent sections.

#### 3.4.1. Total Nitric Oxide and Peroxide in Plasma

**Figure 6:**
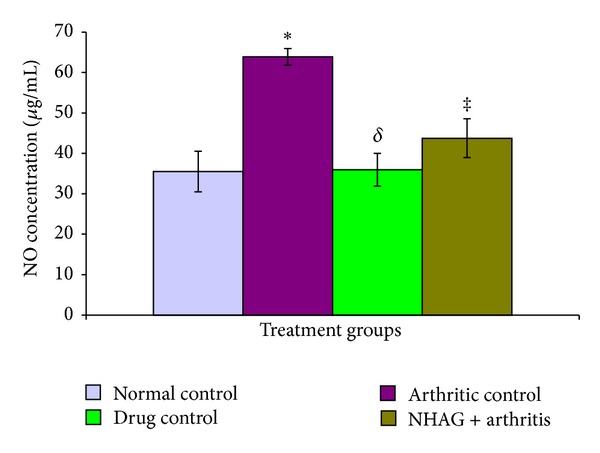
The plasma level of NO determined in the normal and arthritic groups. The arthritic control rats showed a significant increase in the levels of nitric oxide (*P < 0.02) compared to the normal control group. The indomethacin and NHAG treatments in arthritic rats showed a significant reduction in the levels of nitric oxide (^*δ*^ *P* < 0.001 and ^‡^ *P* < 0.02) compared to the arthritic control group, respectively. Within the treatment groups, no significant difference was found.

**Figure 7:**
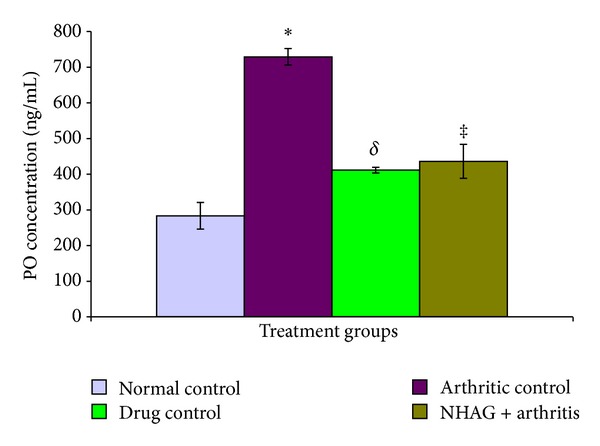
The level of PO analyzed in the plasma samples from the normal and arthritic groups. The arthritic control rats showed a significant increase in the levels of peroxide (*P < 0.002) compared to the normal control group. The indomethacin- and NHAG-treated arthritic rats showed a significant reduction in the levels of peroxide (^*δ*^ *P* < 0.02 and ^‡^ *P* < 0.03) compared to the arthritic control group, respectively. Within the treatment groups, no significant difference was found.

In comparison to the normal control group, a significant increase in the plasma levels of NO (*P* < 0.02) and PO (*P* < 0.002) was observed in case of the arthritic control animals (Figures 6 and 7). In comparison to the arthritic control group, a significant reduction in the levels of NO and PO was found in both the indomethacin- (*P* < 0.001 for NO, *P* < 0.02 for PO) and NHAG- (*P* < 0.02 for NO, *P* < 0.03 for PO) treated groups. Within the treatment groups, no significant difference was found between NHAG treatment and indomethacin treatment. The statistical analysis showed nonsignificant difference between normal and both of the treated group.

#### 3.4.2. Plasma Glutathione and SOD

**Figure 8:**
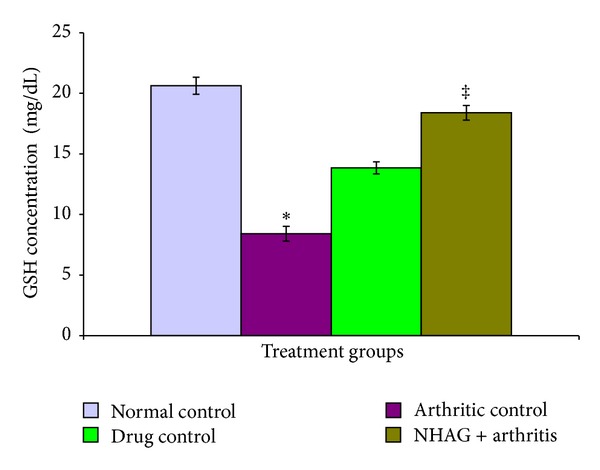
The plasma glutathione (mg/dL) concentration measured in the arthritic and nonarthritic groups. When compared with the normal control group, a significant decrease in the level of glutathione (*P < 0.02) was observed in the arthritic control animals. The NHAG treatment demonstrated a significant rise in the GSH level in comparison to the control arthritic group (^‡^ *P* = 0.04). The treatment of indomethacin (5 mg/kg) did not exhibit any significant effect on the measured parameter.

**Figure 9:**
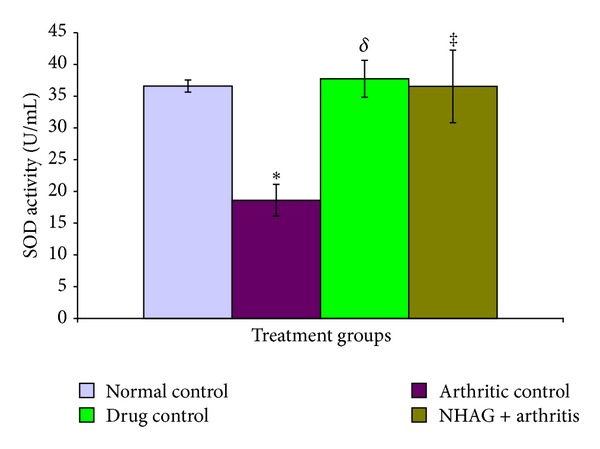
The plasma levels of superoxide dismutase determined in terms of activity in the normal and arthritic groups. The arthritic control rats showed a significant decrease in the levels of SOD activity (*P < 0.02) compared to the normal control group. The indomethacin- and NHAG-treated arthritic rats showed a significant increase in the levels of SOD activity (^*δ*^ *P* < 0.003 and ^‡^ *P* < 0.007) compared to the arthritic control group, respectively. Within the treatment groups, no significant difference was found.

The glutathione concentration in the plasma of arthritic and nonarthritic groups is shown in Figure 8. A significant reduction in the GSH level (*P* < 0.02) of the arthritic animals in comparison to the normal control group was observed. When the arthritic control group was compared with the NHAG- and indomethacin-treated arthritic animals, we found a significant increase in the level of GSH in the NHAG-treated group (*P* < 0.04). Although, higher concentration of GSH was observed in case of indomethacin treatment; however, when the data was statistically analyzed, no significant difference was found compared to the arthritic control group (*P* < 0.19). Next, we compared the treatment groups, that is, indomethacin treatment versus NHAG. The NHAG treatment demonstrated better results in terms of significantly increasing GSH. The activity of SOD measured in the plasma of arthritic control rats was significantly (*P* < 0.02) decreased in comparison to the normal control animals (Figure 9). In contrast, the arthritic rats treated with NHAG and indomethacin showed a marked increase in the level of SOD activity *P* < 0.003 and *P* < 0.007 for indomethacin and NHAG, resp.). When compared to the normal control group, both of the treatments showed a slight but nonsignificant decrease in the SOD activity. 

### 3.5. Proinflammatory Cytokines TNF-*α* and IL-1*β*

**Figure 10:**
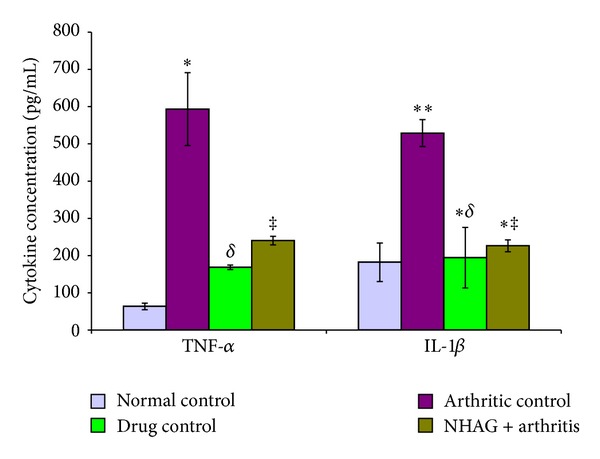
The serum levels of proinflammatory cytokines measured in the arthritic and nonarthritic animals. The arthritic control rats showed a significant increase in the levels TNF-*α* (*P < 0.001) and IL-1*β* (**P < 0.001) compared to the normal control group. The indomethacin- and NHAG-treated arthritic rats showed a significant increase in the levels of TNF-*α* (^*δ*^ *P* < 0.001 and ^‡^ *P* < 0.001) and IL-1*β* (^∗*δ*^ *P* < 0.002 and ^∗‡^ *P* < 0.001) compared to the arthritic control group, respectively. Within the treatment groups, no significant difference was found. The concentration of IL-1*β* in both the treated groups was comparable to normal control. In contrast the TNF-*α* concentration in the treated groups was higher compared to normal control; however, it was found statistically non-significant.

Figure 10 demonstrates the serum levels of IL-1*β* and TNF-*α* in arthritic and nonarthritic animals. It was observed that both IL-1*β* (*P* < 0.001) and TNF-*α* (*P* < 0.001) increased significantly in the serum of arthritic control rats compared to the normal control group (Figure 10). The IL-1*β* and TNF-*α* were found to be significantly decreased in indomethacin- (*P* < 0.002 for IL-1*β*, *P* < 0.001 for TNF-*α*) and NHAG- (*P* < 0.001 for IL-1*β*, *P* < 0.001 TNF-*α*) treated arthritic groups compared to arthritic control group. The comparison within the treatment groups was found to be statistically nonsignificant in case of both IL-1*β* and TNF-*α*. The vehicle that is saline used for solubilizing the NHAG was also tested, and we did not observe any effect of saline on the concentration of the measured cytokines.

## 4. Discussion

In the present study, the female SD rats were used which are considered to be the moderate-responder strain to AIA and demonstrated that following the intradermal inoculation with CFA they developed full blown arthritis at 100% incidence reproducibly. After validating the model, we have used it to study the modulating effects of NHAG on adjuvantinduced arthritic rats. 

The arthritis was examined and graded by arthritic and histological scoring system. The use of both protocols ensures a thorough evaluation of lesions and decreases the likelihood of occult lesions. In the time course of the development of arthritis, two phases were prominent, that is, (1) erythema and local swelling of the paw (acute phase) and (2) systemic disease (chronic phase) that extends to the other paws. We observed that NHAG not only significantly suppressed the paw inflammation associated with the disease, but it also retards the increase in the arthritic score (Figure 2). The observations were almost comparable to that of the reference drug used. 

The quantification of the changes in paw volume as an indicator of oedema was used to monitor the progression of the disease. The paw oedema is due to the infiltration of inflammatory mediators like cytokines, lymphocytes, prostaglandins, and ROS along with release of substance P. The anti-inflammatory effect of the compound was checked in terms of the decrease in the paw volume increment as compared to that of the arthritic control group (Figure 3). We observed a significant increase in the paw volume of the arthritic rats compared to the normal control group which represent inflammatory reaction in response to the induced adjuvant. On the other hand, the indomethacin- and NHAG-treated arthritic rats showed effective reduction in the paw oedema. This represents there anti-inflammatory effect in the AIA model. 

Another indicator of disease progression, the change in body weight of both the treated and untreated arthritic rats, was also measured. The arthritic rats showed a gradual decrease in their body weights until the end of each experiment (Figure 4). In contrast, the NHAG and indomethacin were able to cease the continued loss of body weight in the rats receiving these treatments. The changes in the body weight seen in the arthritic rats treated with indomethacin or NHAG correlate with their effect on inflammation. Various studies in the inflammatory pain model reported that the agents reducing oedema also prevent the inflammatory cells recruitment towards the arthritic joints [36, 37] and hence reverse the limiting movement of the animal and allow free access to the food. Furthermore the glucosamine which is the parental compound of NHAG has also been reported to increase circulating leptin levels in human as well as in rodents [38] that maintain or increase the body weight. Based on these reports and our observations, we suggest that since NHAG is an analogue of glucosamine and also found to reduce the inflammation, therefore, it is inhibiting the secondary hyperalgesia in the same way as that of the glucosamine. 

Pain associated with RA is responsible for disability and the approach of managing arthritis is mostly to relieve the pain. Following arthritic inflammation, Rodent walk with imbalanced gait that become progressively asymmetric over time, thus indicating that animals with joint inflammation or damage spent less time on their injured limb with a slight limp. The gait analysis aids in behavioral assessments and describes functional and symptomatic consequences of knee instability. Any disturbance in gait is considered to be characteristic sign of arthritic pain. Analysis of the gait parameters may be a simple, feasible, and sensitive method to measure arthritis in patients and in arthritic animals.

To measure the nociception associated with the development of arthritis in AIA rats, the gait analysis was employed in our study. In the previous studies the gait analysis is applied to explore the changes in gait associated with the development of arthritis in AIA rats [39–41]. The data reported herein indicate that gait analyses are sensitive to knee joint instability and cartilage remodeling and yield robust comparisons of the affected and contralateral limb. Analyzing gait, we observed that the arthritic control rat exhibited significant changes in all measured gait parameters. The gait of the arthritic control rats was sluggish with significant reduction in both the speed and strides length with parallel increase in the stance and swing time as compared to the normal animals. The results of gait analysis for arthritic control animals in our experiment correlate well with those reported by Coulthard et al. [39] and Simjee et al. [40, 42]. We suggest that altered gait parameters in arthritic rats are because of an increase in the nociception in inflammatory hyperalgesic paws which leads the animals to spend more time with their rare paw in contact with the ground as the mechanical stimuli, thereby decreasing the mean velocity and stride length with parallel increase in the stance and swing time. On the other hand, the treated animals demonstrated a reduction in the paw swelling and gradual increase in the body weight; therefore parallel reversal of the gait deficits seen in them reflects the extent of the pain signals inhibition in the inflamed joints. Thus our results indicate that rodent gait characteristics are capable of tracking the symptomatic consequences of the inflamed joints in the rodent model of arthritis. Based on these reports and our data, we can conclude that treatment with NHAG can be effective in preventing the development of the chronic pain associated with the arthritis.

In addition to macroscopic examination in the experimental animals, the histopathological examination of the arthritic tibiotarsal joints was also performed (Figure 5). We observed a parallel positive relationship between the histological score of arthritis and severity of disease progression. Extensive proliferation of synovial cells, cartilage destruction and infiltration of leukocytes in synovial region were prominent histological features that we observed in the arthritic control samples. Our observations were in correlation with the earlier studies reporting the changes associated with arthritis, that is, proliferation of inflammatory cells with consequent cartilage degradation and erosion [43, 44]. The cartilage destruction is mainly triggered by proinflammatory molecules including leukotrienes, prostaglandins, proteinases, and oxidative species secreted by activated macrophages and fibroblasts in inflamed tissues [45]. The increase removal of osteoclasts from the cartilage as inflammatory response is observable in the form of bone erosion in the histological examination of joints [46, 47]. The treatments which are capable of inhibiting the joint inflammation are considered to be therapeutically efficient for RA [48, 49]. The histological examination of the arthritic joints treated with indomethacin exhibited mild inflammatory cell migration. Since indomethacin is a potent NSAID, it exerts its anti-inflammatory effect through the inhibition of prostaglandins that effectively suppress the inflammatory symptoms in arthritic joints. However, it delivers symptomatic relief, that is, pain and swelling in RA patients but cannot preclude joint destruction [50]. In contrast to indomethacin, NHAG-treated arthritic animals revealed mild increase synovial infiltration with slight bone and periarticular cartilage destruction. It is reported that glucosamine, the parent compound of NHAG, is the basic constituent of glycosaminoglycans in cartilage and synovial fluid. Administration of glucosamine has positive effects in articular cartilage and joint tissue [51, 52]. It enhances the biosynthesis of proteoglycans and inhibits the production of matrix degrading enzymes, that is, collagenases and matrix metalloproteinases from chondrocytes and synoviocytes [53, 54]. We suggest that the NHAG, being a derivative of glucosamine, share common function and retard the damage in articular cartilage and bone. The NHAG suppresses the chronic inflammatory cell infiltration, synovial hyperplasia, and cartilage destruction that correlate to the data obtained from morphological joint scoring.

Based on macroscopic examination of disease progression and histological findings, we suggest that the gait deficits observed in arthritic rats are probably due to inflammatory processes and disease progression which are the major complications of arthritis. Therefore, it is hypothesized that the compound effective in arthritic condition must be able to reverse the gait deficits seen in the arthritic rats. Since both the weight reduction and joint inflammation are seen as the commonly manifested observations in arthritic patients and animals, therefore, we also measured these two parameters to monitor the progression of disease over a time period taken by the control arthritic rats to demonstrate full blown arthritis symptoms. 

The role of reactive oxygen and nitrogen species (ROS and RNS) in arthritis is not surprising since oxidative stress or ROS serves as mediators in pathogenesis of cartilage destruction and tissue damage [55, 56]. ROS-induced cartilage destruction can be inhibited by endogenous SOD or GSH. Imbalance in this mechanism during an aggravated cellular response in arthritis promotes the ROS-induced destruction of bone and cartilage [57, 58]. Our data strongly support these studies in case of arthritic control animals since we have observed a significant rise in the levels of NO and PO with parallel decrease in GSH and SOD. The levels of these measured parameters were also in accord with our histological analysis showing tissue damage and intense immune cells infiltration. 

In the drug control group, that is, indomethacin-treated arthritic animals, we observed a significant reduction in the levels of NO and PO and almost parallel increase in the GSH and SOD (Figures 6–9). Our data is well supported by the studies that reported an inhibitory action of indomethacin on ROS production by inhibiting COX and prostaglandin E_2_ [59, 60]. It has also been reported that increase in the SOD might be related to inhibitory effect of indomethacin on generation of superoxide anions resulting in availability of high levels of SOD to show activity [61]. These studies validate our choice of using indomethacin as a reference drug. We also observed that in comparison to the arthritic control group, NHAG treatment significantly reduces the levels of PO and NO with simultaneous increase in the GSH and SOD activities. The parent compound of NHAG, that is, glucosamine has been studied for it suppressive effects on IL-1*β*-induced NF-*κ*B in chondrocytes which regulate the expression of ROS (iNO) and antioxidants [62–64]. In light of these reports, we suggest that the antioxidant effect of NHAG might be due to its interference with NF-*κ*B pathway and ultimate inhibition of PO and NO.

The immune cells such as activated T-lymphocytes, macrophages, and proliferating synovial cells are the most important contributing factors in pathogenesis arthritis [65]. Studies have reported an increased production of pro-inflammatory cytokines such as IL-1*β*, IL-6, and TNF-*α* by the activated T cells and macrophages [66, 67]. The joint inflammation and cartilage degradation associated with RA are due to an imbalance in pro- and anti-inflammatory cytokines released by mononuclear leukocytes, lymphocytes, and synovial fibroblasts [68]. In the arthritic patient and experimental animals, the significantly high levels of pro-inflammatory cytokines are reported in the circulation and arthritic joints because of the overproliferated inflammatory cells in the articular cavity. The cytokines stimulate synovial fibroblasts and adjacent chondrocytes within the articular cartilage thereby secreting enzymes that degrade proteoglycans, collagen, and connective tissues, resulting in tissue destruction. In accord with these reports, we have also observed significantly high levels of IL-1*β* and TNF-*α* in the arthritic control serum samples (Figure 10). This increase in the inflammatory cytokines also correlates well with the disease progression and supports the histopathological observations measured in case of arthritic control animals. Since these pro-inflammatory cytokines are considered to play a prominent role in the pathogenesis of arthritis and the regulation of these cytokine levels in arthritic subjects is one of the approaches to the treatment of arthritis, therefore we studied the effects of our test compound NHAG on the serum profile of IL-1*β* and TNF-*α*. 

In comparison to the arthritic control, the serum samples from the animals receiving NHAG treatment demonstrated a significant drop in the levels of IL-1*β* and TNF-*α*. The parent compound of NHAG, that is, glucosamine has been extensively studied and reported to exert anti-inflammatory effects by suppressing IL-1*β* and TNF-*α* secretion from macrophages within the inflamed cartilage [69]. Immunomodulatory and chondroprotective effect of glucosamine is also responsible for reversing the joint deterioration associated with arthritis [70, 71]. Based on these reports, we suggest that NHAG might be exerting suppressive effect on IL-1*β* and TNF-*α* by the mechanism similar to glucosamine.

## 5. Conclusion

For the first time, in case of NHAG, we have demonstrated that this compound has potent antirheumatic and anti-inflammatory activities in animal model of arthritis. The present study showed that NHAG treatment significantly inhibited algesia and oedema induced by adjuvant and also decreased the levels of the inflammatory markers, thus establishing that NHAG may be potential candidate for antiarthritic and immunomodulatory activities. 

## Figures and Tables

**Table 1:** Treatment regime followed for the antiarthritic testing of NHAG in the AIA rats.

Groups	Treatment	Doses	Route of administration
GI (*Normal control*,* nonarthritic*)	—	—	—
GII (*Arthritic control*)	—	—	—
GIII (*Drug control*, *Indomethacin + arthritis*)	Indomethacin	5 mg/kg	Intraperitoneal (i.p.)
GIV (*NHAG *+* arthritis*)	NHAG	5 mg/kg	Intraperitoneal (i.p.)

**Table 2:** Individual gait parameters measured on day 0 and day 20. The values are expressed as the mean ± SEM (*n* = 12), where (*) designates (*P* < 0.02) compared to arthritic control animals.

Treatment Group	Day	Speed (cm/sec)	Stride length (cm)	Stance time (s)	Swing time (s)
Normal control	0	24.16 ± 0.37	15.05 ± 0.26	5.40 ± 0.01	2.89 ± 0.005
	20	30.66 ± 0.72*	15.11 ± 0.24*	4.93 ± 0.01*	2.49 ± 0.005*
Arthritic control	0	25.28 ± 0.99	14.24 ± 0.48	5.54 ± 0.02	2.59 ± 0.021
	20	19.31 ± 1.04	10.68 ± 0.44	7.64 ± 0.02	4.43 ± 0.025
Drug control (5 mg/kg)	0	26.09 ± 1.0	12.76 ± 0.77	5.03 ± 0.01	2.99 ± 0.019
	20	23.69 ± 0.54*	13.41 ± 0.28*	6.23 ± 0.03*	3.25 ± 0.008*
NHAG + arthritis (5 mg/kg)	0	23.53 ± 0.49	12.73 ± 0.43	5.29 ± 0.01	3.07 ± 0.018
	20	24.01 ± 0.66*	12.76 ± 0.11*	6.52 ± 0.03*	2.89 ± 0.006*
